# Peculiarities of the course of mental disorders after the coronavirus disease COVID-19 in patients with a history of mental disturbances

**DOI:** 10.1192/j.eurpsy.2025.1274

**Published:** 2025-08-26

**Authors:** N. O. Maruta, V. Y. Fedchenko

**Affiliations:** 1 Borderline Psychiatry, “Institute of Neurology, Psychiatry and Narcology named after P.V. Voloshyn of NAMS of Ukraine” SI, Kharkiv, Ukraine

## Abstract

**Introduction:**

The psychopathological consequences of the complex impact of the coronavirus disease COVID-19 become the basis for the deterioration of the mental state of persons with a history of mental disturbances.

**Objectives:**

To investigate the impact of the clinical and psychopathological consequences of COVID-19 on the course of mental disorders in patients with a history of mental disturbances.

**Methods:**

95 patients with a history of mental disorders who have experienced COVID-19 were examined and made up the main group (F 32.0-32.2, 33.1, 33.2 – 31 patients, F 06.3, 06.4 – 33 patients, F 41.1, 41.2, 42.2, 45.3, 48.0 – 31 patients). The comparison group included patients with 3 or more episodes of mental disorders in the anamnesis, including the current one. Clinical-psychopathological, clinical-amnestic, psychometric (the Clinical Global Impression Scale (CGI)) and methods of statistical analysis were applied.

**Results:**

A comparison of the course dynamics of current mental disorders after COVID-19 and previous mental disturbances in the anamnesis of the studied patients was carried out according to the indicators of the duration and severity of mental disorders, as well as the duration of the remission that preceded them. The duration of current mental disorders after COVID-19 in the examined patients in most cases ranged from 2 weeks to 6 months (46.32% of persons); duration of remission preceding mental disorders after COVID-19 – from 6 to 12 months (47.37% of persons); the initial degree of severity of psychopathological manifestations according to the CGI-S scale – as “moderate disorder” (36.84% of persons). In comparison with the duration of remission preceding mental disorders in the anamnesis, it was found that in significantly more patients, the duration of remission preceding COVID-19 corresponds to the shortest interval from 6 to 12 months (р < 0.05). When comparing with the severity of mental disorders preceding COVID-19, it was established that a “pronounced disorder” was detected in significantly more patients after COVID-19 (p < 0.05). In comparison with the duration of mental disorders preceding COVID-19, no significantly difference was found, however, when comparing indicators of minimum duration from 2 weeks to 6 months (46.32% of cases with current and 58.18% with previous disorders), the reliability indicator was p = 0.0510.

**Conclusions:**

The obtained data indirectly confirm the complex impact of the SARS-CoV-2 pandemic on the formation and exacerbation of mental disorders, indicate a tendency to increase the severity of mental disorders as a result of the coronavirus disease, and also give reason to put forward a hypothesis about an increase in the duration of mental disorders after COVID-19, which is likely will appear on samples with a larger number of observations.

**Disclosure of Interest:**

None Declared

